# Biallelic variants in the *COQ4* gene caused hereditary spastic paraplegia predominant phenotype

**DOI:** 10.1111/cns.14529

**Published:** 2023-11-27

**Authors:** Qiao Wei, Hao Yu, Pei‐Shan Wang, Juan‐Juan Xie, Hai‐Lin Dong, Zhi‐Ying Wu, Hong‐Fu Li

**Affiliations:** ^1^ Department of Medical Genetics and Center for Rare Diseases, and Department of Neurology in Second Affiliated Hospital, and Key Laboratory of Medical Neurobiology of Zhejiang Province Zhejiang University School of Medicine Hangzhou Zhejiang China; ^2^ Nanhu Brain‐computer Interface Institute Hangzhou China

**Keywords:** Chinese, *COQ4*, hereditary spastic paraplegia, novel phenotype

## Abstract

**Introduction:**

Hereditary spastic paraplegias (HSPs) comprise a group of neurodegenerative disorders characterized by progressive degeneration of upper motor neurons. Homozygous or compound heterozygous variants in *COQ4* have been reported to cause primary CoQ10 deficiency‐7 (COQ10D7), which is a mitochondrial disease.

**Aims:**

We aimed to screened *COQ4* variants in a cohort of HSP patients.

**Methods:**

A total of 87 genetically unidentified HSP index patients and their available family members were recruited. Whole exome sequencing (WES) was performed in all probands. Functional studies were performed to identify the pathogenicity of those uncertain significance variants.

**Results:**

In this study, five different *COQ4* variants were identified in three Chinese HSP pedigrees and two variants were novel, c.87dupT (p.Arg30*), c.304C>T (p.Arg102Cys). More importantly, we firstly described two early‐onset pure HSP caused by *COQ4* variants. Functional studies in patient‐derived fibroblast lines revealed a reduction cellular CoQ10 levels and the abnormal mitochondrial structure.

**Conclusions:**

Our findings revealed that bilateral variants in the *COQ4* gene caused HSP predominant phenotype, expanding the phenotypic spectrum of the COQ4‐related disorders.

## Graphical abstract

Five *COQ4* variants were identified in three Chinese HSP pedigrees. Functional studies revealed reduced CoQ10 level and fragmented mitochondria in patient's fibroblast.

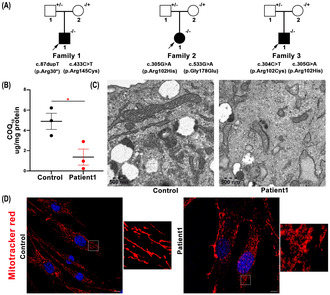

## 1 INTRODUCTION

Hereditary spastic paraplegias (HSPs) are a group of clinically and genetically heterogeneous neurodegenerative disorders.^1^ The critical manifestations are muscle weakness and spasticity in lower extremity, which may appear in isolation (pure HSP), or be accompanied by other symptoms (complicated HSP), such as peripheral neuropathy, intellectual dysfunction, cerebellar ataxia, and seizures.^2^, ^3^ Due to the heterogeneous features of HSP, it is overwhelmingly difficult to distinguish HSP from other neurogenetic diseases, such as mitochondrial diseases.^4^ In recent years, more and more novel genes involved in HSP have been detected by using whole exome sequencing (WES) and whole genome sequencing (WGS). To date, HSP can be divided into many subtypes (SPG1‐88) and caused by variants in at least 70 genes, which include almost all inheritance patterns, namely autosomal dominant (AD), autosomal recessive (AR), X‐linked recessive (XLR), and mitochondrial.^5^ However, many cases of pure or complicated HSP still remain unexplained.

COQ4, encoded by *COQ4*, is one of coenzyme Q10 (CoQ10) subunits and mainly localized to the matrix side of the mitochondrial inner membrane.^6^ Pathogenic variants in *COQ4* (MIM 612898) are the cause of autosomal recessive primary CoQ10 deficiency‐7 (COQ10D7), first identified in 2015.^7^ The COQ10D7 has an extremely broad phenotypic spectrum, manifesting as lethal neonatal‐onset mitochondrial encephalopathy, developmental delay, cognitive defects, epilepsy, childhood‐onset ataxia, visual dysfunction, cardiomyopathy, and respiratory insufficiency.^8^ Some of the clinical features of COQ10D7 overlap with those of HSP.

In this study, we first reported two patients with clinical features of pure HSP caused by biallelic pathogenic variants in the *COQ4* gene.

## 2 MATERIALS AND METHODS

### 2.1 Subjects

We recruited 87 genetically unidentified HSP index patients and their available family members from the Department of Neurology in the Second Affiliated Hospital of Zhejiang University School of Medicine between August 2015 and January 2023.^9^, ^10^, ^11^ All patients were diagnosed based on Harding's criteria and evaluated by at least two experienced neurologists. All patients present with progressive spastic paraplegia, with or without associated features, and were excluded from acquired causes. Most of the probands underwent biochemical tests, magnetic resonance imaging (MRI) and electromyogram (EMG), the flow chart was seen in Figure S1. This study was approved by the local Ethics Committee and the written informed consent was obtained from each participant. In addition, 1500 healthy controls were involved in this study.

### 2.2 Genetic analyses

Peripheral blood was collected from all participants, and genomic DNA was isolated by using QIAamp blood genomic extraction kits (Qiagen, Hilden, Germany). WES was performed in all probands using Agilent SureSelect Human All Exon v6 kit on the Illumina HiSeq X Ten platform (XY Biotechnology Co. Ltd, Hangzhou, China). Variants with minor allele frequency (MAF) of <1% in the Genome Aggregation Database (gnomAD), Exome Aggregation Consortium (ExAC) Browser, and the 1000 Genomes Project database (1000G) were included. Software programs (SIFT, PolyPhen‐2, Mutation Taster, M‐CAP, CADD, and REVEL) were performed to estimate whether a variant change protein structure or function. Sanger sequencing was performed to confirm probable variants, and co‐segregation analysis was further carried out in available family members. Pathogenicity of variant was subsequently classified according to the American College of Medical Genetics and Genomics (ACMG) standards and guidelines.^12^

### 2.3 Copy number variation analysis and Multiplex ligation‐dependent probe amplification assay

Copy number variations (CNVs) were detected from WES data as previous description.^11^ Patients remaining negative following WES were further screening for large deletions or duplications of *SPAST*, *ATL1*, *REEP1*, *SPG7*, and *SPG11* by the MLPA analysis, using commercially available MLPA kits (SALSA P165‐C2 for *SPAST* and *ATL1*; SALSA P213‐B2 for *REEP1* and *SPG7*; SALSA P306‐B1 for *SPG11*. MRC‐Holland, the Netherlands) according to the manufacturer's recommendations.

### 2.4 RNA isolation and quantitative real‐time PCR (qPCR)

Total RNA was extracted from the peripheral blood samples. Synthesis of total cDNA was carried out using a PrimeScript RT reagent kit (Takara) according to the manufacturer's protocol. We performed Quantitative real‐time PCR (qPCR) experiments using a SYBR Premix Ex Taq Kit (Takara) and an ABI StepOnePlus sequence detection system (Thermo Fisher Scientific). The primer sequences are were as follows: COQ4‐F: GATGGCGCTCTATAACCCCTA; COQ4‐R: GGTGGATGTCGAAATCCGGG; GAPDH‐F: ACTCCACGACGTACTCAG; GAPDH‐R: CATGTTCCAATATGATT.

### 2.5 Plasmid constructs

The coding sequence of the human wild‐type (WT) *COQ4* gene (NM_016035.5) were cloned into pcDNA3.1‐3xFlag‐C vector. The variants within *COQ4* (p.Arg102Cys, p.Arg145Cys, p.Arg145Gly) were introduced to plasmids using PCR mutagenesis.

### 2.6 Cell culture and transfection

HEK 293T cells were cultured in DMEM (HyClone) supplemented with 10% fetal bovine serum (Gibco) at 37°C under 5% CO_2._ Then cell lines transiently transfected with WT or mutant plasmids using Lipofectamine 3000 reagent (Invitrogen) and were collected for further analysis 48 h later.

We obtained the biological samples from one healthy control and the proband of family 1 using a 4‐mm skin biopsy. Each participant signed written informed consent. Then we established the primary fibroblast cell lines. Fibroblasts were grown in Dulbecco's modified Eagle's medium (Gibco) supplemented with 10% fetal bovine serum (Gibco) and 1% penicillin/streptomycin (Gibco) at 37°C in 5% CO_2_.

### 2.7 Western blot analysis

Protein samples from HEK 293T cells were resolved by 10% SDS‐polyacrylamide gel electrophoresis (SDS‐PAGE) and then transferred to Polyvinylidene fluoride (PVDF) membranes. Specific bands were detected with anti‐flag (1:1000) (Abmart), anti‐GAPDH (1:5000) (Abmart), respectively. Quantification of density in each band was performed by Image‐J software (NIH).

### 2.8 Immunofluorescence analysis

Fibroblast cells were cultured in glass‐bottomed dishes. The mitochondria was visualized using MitoTracker red probe (Invitrogen). Then cells were washed with ice‐cold phosphate‐buffered saline (PBS) three times and were fixed with 4% paraformaldehyde for 15 min at room temperature. Fluorescence images were captured by ZEISS LSM900 confocal system.

#### 2.8.1 Measurement of CoQ10 content

Fibroblast cell lines were cultured and lipid samples was extracted by using 3 mL n‐hexane. Levels of CoQ10 were measured using ultra‐performance liquid chromatography‐electrospray ionization tandem mass spectrometry (UPLC‐ESI‐MS/MS) analysis (Agilent HPLC‐1200).

### 2.9 Transmission electron microscope

Fibroblasts were cultured on 15 cm dishes and then harvested by 0.25% trypsinization. We used 2.5% glutaraldehyde to fix fibroblasts. All samples were washed for 10 min three times with 1XPBS, post‐fixed in 1% osmium solution, and then embedded in epoxy resin. Images were obtained by using H7500 transmission electron microscope (TEM) (Hitachi).

### 2.10 Statistical analysis

Data are presented in the figures as the mean ± SD. Statistical analyses were performed using GraphPad Prism (GraphPad Software for Mac). Statistical significance was assessed by two‐tailed Student's *t*‐test and one‐way analysis of variance (ANOVA) test for comparison of two groups. Multiple comparisons within the group were tested against the corresponding control. Significance levels are indicated in the figure legends. Differences were considered statistically significant when *p < 0.05, **p < 0.01, ***p < 0.001, or ****p < 0.0001.

## 3 RESULTS

### 3.1 Genetic findings

**FIGURE 1:**
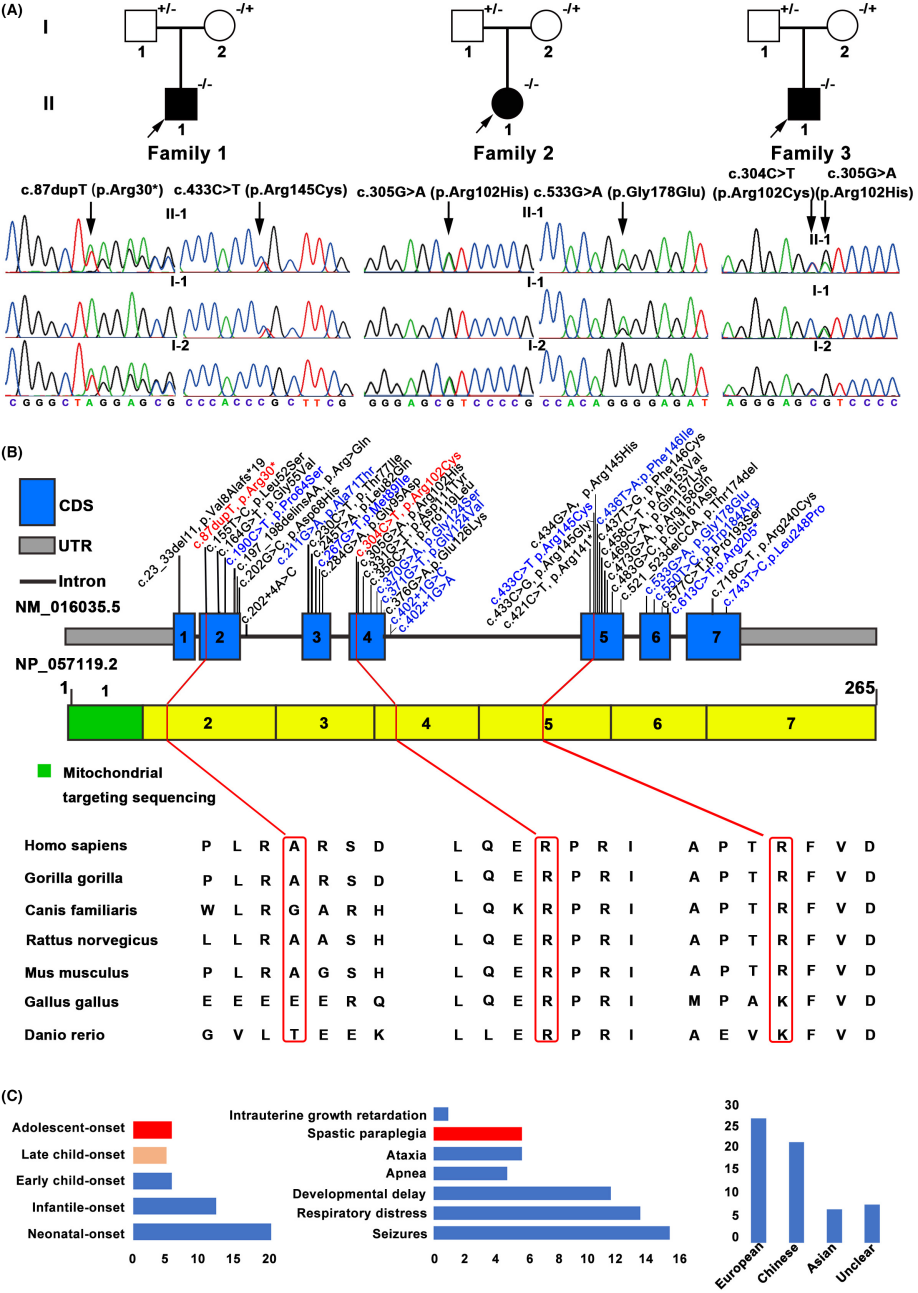
Pedigree, sequencing electropherograms, localizations and conservation variant in *COQ4*. (A) Pedigree and Sanger sequencing data of variants within *COQ4* gene of the family. Square indicates male; circle indicates female; the black symbol indicates affected individual; arrow indicates the proband. Symbol with “−/−” indicates patient. Symbol with “+/−” indicates mutation carrier. (B) *COQ4* structure and amino acids evolutionary conservation of amino acids. All the known *COQ4* variants are reported on the transcript; novel variants are in red and variants detected in Chinese patients are in blue. Conservation of the residues surrounding arginine (Ala, A) 30; (Arg, R) 102; (Arg, R) 145 among species. (C) Phenotypic spectrum of COQ4 deficiency of 65 patients, including 3 newly described patients. Age at first presentation and the red bar represents all of our patients, while the orange bar is part of our patients (left); Symptoms at onset and the red bar represents all of our patients (middle); Ethnicity of patients (right).

WES analysis was performed in 87 genetically unidentified HSP index probands. After filtering, five variants in *COQ4* gene (NM_016035.5) were identified in three index patients (Figure 1A and Table 1). Two variants (c.87dupT: p.Arg30*, c.304C>T: p.Arg102Cys) were novel and three (c.305G>A: p.Arg102His, c.433C>T:p.Arg145Cys, c.533G>A: p.Gly178Glu) were previously reported.^13^, ^14^ Two novel variants had an extremely low frequency in the public database, including 1000G, ExAC, gnomAD, and absent in our 1500 in‐house WES database. Two missense variants (p.Arg145Cys, p.Arg102Cys) were in the same amino acid position as previously identified pathogenic variants p.Arg145Gly and p.Arg102His, respectively. These two variants were predicted as deleterious variants using five in silico software (SIFT, PolyPhen‐2, Mutation Taster, M‐CAP, CADD, and REVEL), and the substitutions were evolutionarily highly conserved (Figure 1B). According to the ACMG standards and guidelines, three novel variants were classified as “pathogenic.” Detailed information about these variants were listed in Table S1.

**TABLE 1:** Clinical features of patients with *COQ4* variants.

Patient	Gender	AAO (y)	Duration (y)	Variant	Phenotype	Spastic gait	UL/LL hyperreflexia	Muscle tone	Babinski sign	Seizures	visual impairment	MRI findings
Patient1	M	6	9	p.Arg30* (Het)*/p.Arg145Cys(Het)*	Pure	+	+++ ++++	Increased	+	−	−	Thin thoracic spinal cord
Patient2	F	17	10	p.Arg102His (Het)/p.Gly178Glu (Het)	Complicated	+	++ ++++	Increased	+	+	+	Bilateral hippocampal sclerosis
Patient3	M	12	3	p.Arg102His (Het)/p.Arg102Cys (Het)*	Pure	+	+++ ++++	Increased	+	−	−	Normal

Abbreviations: *, novel variant; AAO, age at onset; F, female; Het, heterozygous; LL, lower limb; M, male; UL, upper limb; y, years.

### 3.2 Clinical features

**FIGURE 2:**
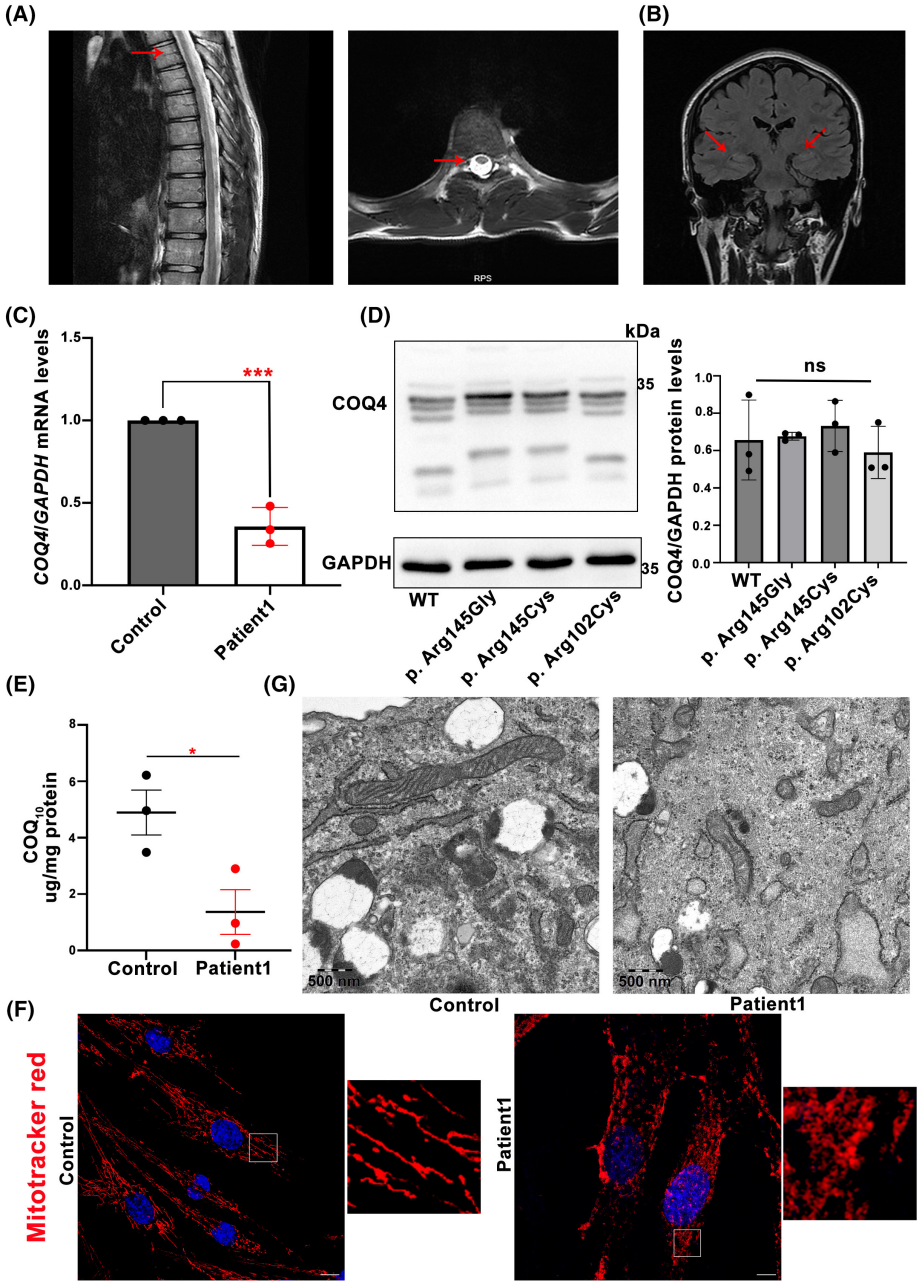
Image data of patients carrying variants in COQ4 gene and functional studies. (A) Spinal MRI of patient 1. (B) Brian MRI of patient 2. (C) Quantitative real‐time PCR analysis of *COQ4* mRNA levels in peripheral blood. (D) Western blot analysis of WT and mutant COQ4 protein level. Three replicates. (E) Quantification of the level of cellular CoQ_10_ by using UPLC‐ESI‐MS/MS analysis. The result shows that patient‐derived fibroblasts show a reduction in CoQ_10_ levels (mean ± SEM; *p < 0.05 significantly different compared from controls based on Student's *t*‐test). (F) Immunofluorescence in fibroblasts derived from patient 1 and control labeled with mitochondria (red) and DAPI (blue). Insets in the images are enlarged (original magnification, 10×). All images were taken by using a 63× objective lens ZEISS LSM900 confocal system. Scale bar, 1 μm. (G) Abnormal structure of mitochondria in patient‐derived fibroblasts. Scale bar, 500 nm.

Patient 1 (II‐1 in Family 1) is a 15‐year‐old male, carrying a novel truncating variant p.Arg30* and a heterozygous missense variant p.Arg145Cys (Figure 1A). His birth history was normal for a full‐term delivery and he also showed normal development for his age. At the age of 6, he was noticed to have abnormal walking posture and a tendency to fall down. As the gait abnormality progressed, he showed difficulty walking and by the age of 15 had lost the ability to walk with ambulation. No seizures, visual impairment, or cognitive deficits were reported. On the physical examinations, dysarthria and representative pyramidal signs (increased muscle tone, hyperreflexia in all limbs, ankle clonus, patellar clonus) were observed. Muscle strength was normal. Brain MRI was normal while spinal MRI showed thin thoracic spinal cord (Figure 2A). Neural conduction velocity and electromyography (EMG) were normal. By the time he was 13, baclofen was prescribed at a dose of 15 milligrams three times a day, and the muscle spasms had been eased. Oral CoQ10 supplementation (150 mg/day) was immediately started after the genetic diagnosis at age 15. However, his clinical status has not improved. His father carried heterozygous p.Arg145Cys and his mother carried heterozygous p.Arg30*.

Patient 2 (II‐1 in Family 2) carrying two reported heterozygous pathogenic variants (p.Arg102His and p.Gly178Glu) is a 27‐year‐old female (Figure 1A). She was the only child of healthy, unrelated parents and was born after an uneventful pregnancy. She showed normal motor and psychological milestones. Her problems began at the age of 17 when her parents noticed postural instability but no difficulty walking. At the age of 22, she developed recurrent seizures, and as her symptoms progressed, she developed dysarthria within a year. At the age of 27, she developed visual impairment. Cognitive ability is not impaired. Slurred speech, spastic gait, increased muscle tone in all limbs, hyperreflexia in the lower limbs, and bilateral extensor plantar response were observed. The brain MRI showed bilateral hippocampal sclerosis and whole spine MRI revealed no abnormalities (Figure 2B). The electroencephalogram (EEG) showed that the high‐amplitude three‐phase cusp wave is emitted at a long distance, and the front part of the head is slightly scratched. Her father is heterozygous for p.Gly178Glu and her mother is heterozygous for p.Arg102His.

Patient 3 (II‐1 in Family 3) was an 18‐year‐old boy affected with HSP, which had started 3 years earlier (Figure 1A). He carried a reported heterozygous pathogenic variant, p.Arg102His and a novel missense heterozygous variant, p.Arg102Cys. The pregnancy was uncomplicated and the developmental milestones were unremarkable. He experienced walking stiffness and a scissor gait by the age of 15. His concentration and learning ability were comparable to those of his peers. There was no dysarthria, dysphagia, cognitive impairment, or visual impairment. Neurological examination revealed increased muscle tone and enhanced brisk patellar reflexes in the lower limbs. The Hoffmann's sign was also presented. Muscle strength and sensation were normal. Other examinations, including cerebellar and sensory tests, showed no abnormalities. Brain MRI, spinal MRI, and EMG were all normal. His father carried heterozygous p.Arg102His, while his mother carried heterozygous p.Arg102Cys.

### 3.3 Biochemical analyses

The expression of COQ4 mRNA in peripheral blood was measured by qPCR analysis, and we found that the mRNA levels were reduced in patient 1 compared to healthy controls (Figure 2C). To clarify the biological effects of two variants (p.Arg145Cys, p.Arg102Cys), the plasmids were constructed and the COQ4 protein level was measured by Western blotting. The results showed that there was no change in COQ4 protein levels compared to the wild type (Figure 2D). We use UPLC‐ESI‐MS/MS to detect the level of cellular CoQ_10,_ levels and quantification analysis showed a significant reduction of CoQ_10_ levels in patient‐derived fibroblast cell lines (Figure 2E). To estimate the mitochondrial distribution pattern, immunofluorescence analysis was performed on the mitochondria from control and patient‐derived fibroblast lines. Mitochondrial network of patient‐derived fibroblasts was more fragmented, and a larger number of short mitochondrial could be observed (Figure 2F). Transmission electron microscopy (TEM) was used to observe the structure of mitochondria. Obvious damage of mitochondrial ultrastructure including the disruption of the mitochondrial membrane and abnormal cristae observed in fibroblasts from Patient 1 (Figure 2G).

## 4 DISCUSSION

In recent years, a variety of new complex spastic paraplegias, particularly with autosomal recessive inheritance patterns, have been described. Complicated HSP can be associated with cerebellar dysfunction, epilepsy, mental retardation, myopathic features, visual abnormalities, and microcephaly.^5^ The overlap between HSP and other inherited diseases, such as mitochondrial disorders, is increasingly recognized. In addition, a range of overlapping syndromes of HSP are often caused by specific genes. This study identified three patients who presented as HSP‐carrying compound heterozygous variants in the *COQ4* gene.

CoQ10 is a lipophilic molecule, mostly located in the cellular mitochondrial inner membrane.^6^ The most important role of CoQ10 is as an electron carrier, which can shuttle electrons derived from complexes I (NADH: ubiquinone oxidoreductase) and II (succinate dehydrogenase) to complex III (ubiquinone‐cytochrome c oxidoreductase). It is also reported to be involved in several other essential biological processes, such as biosynthesis of pyrimidine, antioxidant response, regulation of the mitochondrial structural stabilization, β‐oxidation of fatty acids, apoptosis, and so on.^8^ So far, there are more than 10 genes directly related to biosynthesis of CoQ10 (*ADCK3*, *PDSS1*, *PDSS2*, *COQ2*, *COQ3*, *COQ4*, *COQ5*, *COQ6*, *COQ7*, *COQ8A*, *COQ8B*, *COQ9*, *COQ10A*, and *COQ10B*). Variants in these genes are associated with primary CoQ10 deficiency, a series of rare but clinically heterogeneous diseases, presenting as multisystem manifestations. There are four major clinical manifestations, an encephalomyopathic form with seizures and ataxia; a multisystemic infantile form with encephalopathy, cardiomyopathy, and renal failure; a predominantly cerebellar form with ataxia and cerebellar atrophy; and Leigh syndrome with growth retardation.^15^ Among the primary CoQ10 deficiency‐related genes, *COQ4* is relatively newly identified. To date, 62 COQ10D7 patients including 21 Chinese patients presenting various clinical phenotypes and 39 variants have been described worldwide by WES.^8^, ^16^, ^17^

In addition, about half of the cases had neonatal‐onset and presented encephalomyopathic form with seizures (Figure 1C). *COQ4* c.370G>A (p.Gly124Ser) is a founder variant in the southern Chinese, and 16 of 21 Chinese patients (88%) carried this variant (9 were homozygous and 7 were heterozygous), indicating a higher chance of occurrence in Chinese. All Chinese patients presented neonatal‐onset or infantile‐onset multisystem symptoms, including severe lactic acidosis, respiratory failure or distress, hypotonia, and cardiomyopathy (Table S2).^13^, ^18^, ^19^, ^20^, ^21^, ^22^ The precise function of COQ4 remains unknown and the current number of reported patients is limited, so there is an urgent need for more and more patients with different clinical phenotypes to aid clinical diagnosis. Here, we described three patients with variants in *COQ4* gene that primarily presented as childhood or adolescent‐onset spastic paraplegia, in contrast to severe cardiac or neurologic symptoms that develop soon after birth and often result in death. It is worth mentioning that two male patients presented with isolated HSP, a novel phenotype for *COQ4*‐related disorders.

In conclusion, we identified five different *COQ4* variants in HSP patients from three different Chinese families. Among them, two variants p.Arg30*and p.Arg102Cys were novel. It is worth noting that we have first described the pure HSP caused by *COQ4* variants. Our results expand the clinical and genetic spectrum of *COQ4*. Therefore, we suggest that primary CoQ10 deficiency‐related genes should be considered when screening the genetic cause of HSP patients.

## AUTHOR CONTRIBUTIONS

Qiao Wei and Hao Yu: Data collection and analysis, functional experiments, Writing and editing. Pei‐Shan Wang, Juan‐Juan Xie, Hai‐Lin Dong: Data collection and analysis. Zhi‐Ying Wu and Hong‐Fu Li: Conceptualization, Wring and editing, Funding acquisition.

## CONFLICT OF INTEREST STATEMENT

The authors declare that they have no known competing financial interests or personal relationships that could have appeared to influence the work reported in this paper.

## Supporting information


Figure S1.

Table S1.

Table S2.

## Data Availability

The data that support the findings of this study are available from the corresponding author upon reasonable request.
