# Supplementary figures and images for: Biallelic variants in the COQ4 gene caused hereditary spastic paraplegia predominant phenotype
Source: CNS Neurosci Ther. 2023 Nov 27;30(4):e14529. doi: 10.1111/cns.14529 (PMC11017416; doi:10.1111/cns.14529)

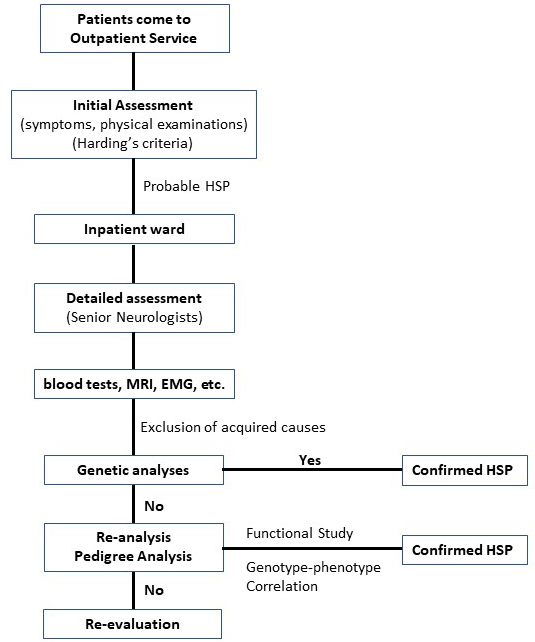

Supplement: Supplementary file 1 — Figure S1. [file CNS-30-e14529-s002.jpg]
