# Supplementary material for: Biallelic variants in the COQ4 gene caused hereditary spastic paraplegia predominant phenotype
Source: CNS Neurosci Ther. 2023 Nov 27;30(4):e14529. doi: 10.1111/cns.14529 (PMC11017416; doi:10.1111/cns.14529)
Supplement: Supplementary file 2 — Table S1. [file CNS-30-e14529-s003.docx]

Table S1**.** Features of pathogenic variants within *COQ4* gene

| Nucleotide  change | Amino acid  change | Exon | 1000G | ExAC | gnomAD | SIFT | | Polyphen-2 | Mutation Taster | M-CAP | CADD | REVEL | ACMG |
| --- | --- | --- | --- | --- | --- | --- | --- | --- | --- | --- | --- | --- | --- |
| c.87dupT | p.Arg30* | 2 | 0 | 0 | 0 | NA | NA | | NA | NA | NA | NA | PVS1,PS4,PM2,PP1, PP2, PP3 |
| c.304C>T | p.Arg102Cys | 4 | 0 | 8.292e^-06^ | 2.442e^-05^ | Damaging | Probably damaging | | Disease causing | Damaging | Damaging | Damaging | PS4,PM1,PM2,PM5, PP1, PP2, PP3 |
| c.433C>T* | p.Arg145Cys | 5 | 0 | 2.634e^-05^ | 9.502e^-06^ | Damaging | Probably damaging | | Disease causing | Damaging | Damaging | Damaging | PS4,PM2,PM5,PP1, PP2, PP3 |

NA, not available; *, this variant hasn’t been published in HGMD.
