# Supplementary material for: Biallelic variants in the COQ4 gene caused hereditary spastic paraplegia predominant phenotype
Source: CNS Neurosci Ther. 2023 Nov 27;30(4):e14529. doi: 10.1111/cns.14529 (PMC11017416; doi:10.1111/cns.14529)
Supplement: Supplementary file 3 — Table S2. [file CNS-30-e14529-s001.docx]

Table S2. Summary of clinical, laboratory, and imaging findings of the reported patients

| Phenotype published | Sex | Allele 1 | Allele 2 | Age at onset | Delayed motor development | Delayed cognitive development | Spasticity | Seizures | Respiratory distress | Visual impairment | Cardiomyopathy |
| --- | --- | --- | --- | --- | --- | --- | --- | --- | --- | --- | --- |
| Yu et al. 2019; 1 | m | c.370G>A | c.402+1G>C | 7 d | yes | yes |  | yes | yes |  | yes |
| Yu et al. 2019; 2 | m | c.370G>A | c.402+1G>C | at birth | yes | yes |  |  | yes |  | yes |
| Yu et al. 2019; 3 | f | c.370G>A | c.370G>A | at birth | yes | yes |  | yes | yes |  | yes |
| Yu et al. 2019; 4 | f | c.370G>A | c.402+1G>C | at birth | yes | yes |  | yes | yes |  | yes |
| Yu et al. 2019; 5 | f | c.370G>A | c.402+1G>C | at birth | yes | yes |  | yes | yes |  | yes |
| Yu et al. 2019; 6 | m | c.550T>C | c.402+1G>A | 8 m | yes | yes |  |  |  | yes |  |
| Yu et al. 2019; 7 | f | c.370G>A | c.370G>A | at birth | yes | yes | yes |  |  | yes |  |
| Yu et al. 2019; 8 | f | c.370G>A | c.371G>T | 6 m | yes | yes | yes | yes |  | yes |  |
| Yu et al. 2019; 9 | m | c.370G>A | c.370G>A | 2 m | yes | yes |  | yes |  | no |  |
| Yu et al. 2019; 10 | f | c.370G>A | c.370G>A | at birth | yes | yes |  | yes | yes | yes | yes |
| Yu et al. 2019; 11 | f | c.370G>A | c.370G>A | 4 m | yes | yes | yes | yes |  |  | yes |
| Lu et al. 2019, II-1 | m | c.370G>A | c.370G>A | at birth | yes | yes |  | yes | yes |  |  |
| Lu et al. 2019 | f | c.370G>A | c.370G>A | at birth | yes | yes |  | yes | yes | yes |  |
| Ling et al.2019; 1 | m | c.370G>A | c.370G>A | 1 m | yes |  |  | yes | yes |  | yes |
| Ling et al.2019; 3 | M | c.370G>A | c.533G > A | Before birth |  |  |  |  | yes |  | yes |
| Chen et al. 2020 | M | c.370G>A | c.370G>A | 1 m | yes |  |  | yes |  | yes |  |
| Ge et al. 2019 | m | c.211G>A | c.436T>A | At birth | yes | yes |  | yes | yes | yes | yes |
| Wu et al. 2022 | - | c.613C>T | c.433C>T | - | - | - | - | - | - | - | - |
| Wu et al. 2022 | - | c.550T>C | c.743T>C | - | - | - | - | - | - | - | - |
| Wu et al. 2022 | - | c.190C>T | c.533G>A | - | - | - | - | - | - | - | - |
| Wu et al. 2022 | - | c.267G>T | c.370G>A | - | - | - | - | - | - | - | - |

f=female; m= male; d= day; m= month; -, Not described.
